# Systemic inflammation mediates the association between the dietary index for gut microbiota and serum Klotho: evidence from NHANES 2007–2016

**DOI:** 10.3389/fnut.2025.1599971

**Published:** 2025-09-03

**Authors:** Xintao Lv, Heng Liu, Yu Zhou, Haidong Hao, Yutang Yuan, Hongtao Jia, Mingchu Jin

**Affiliations:** Department of Urology, Renmin Hospital, Hubei University of Medicine, Shiyan, China

**Keywords:** gut microbiota, Klotho, SII, SIRI, NHANES

## Abstract

**Background:**

The Dietary Index for Gut Microbiota (DI-GM) is a novel dietary assessment tool reflecting the impact of diet on gut microbiota composition. Serum Klotho, an anti-aging protein, has been implicated in various metabolic and inflammatory processes. However, the relationship between the DI-GM Score and serum Klotho levels, as well as the potential mediating role of inflammatory markers, remains unclear. This study aims to investigate the association between DI-GM and serum Klotho and to explore the mediating effect of systemic inflammation.

**Methods:**

This cross-sectional study utilized data from the 2007–2016 National Health and Nutrition Examination Survey (NHANES). Participants aged 40–79 years were included, and a total of 12,341 individuals remained after applying exclusion criteria. The DI-GM Score was calculated based on dietary intake data, while serum Klotho levels were measured using ELISA. The systemic immune-inflammation index (SII) and systemic inflammation response index (SIRI) were derived from complete blood counts to assess inflammation. Multivariate linear regression models, restricted cubic spline (RCS) analysis, subgroup analysis, and mediation analysis.

**Results:**

DI-GM Score was positively associated with serum Klotho levels after adjusting for confounders (*β* = 4.05, 95% CI: 1.31, 6.80, *p* = 0.004). Compared to participants with the lowest DI-GM scores (0–3), those with DI-GM ≥ 6 had significantly higher serum Klotho levels (*β* = 21.65, 95% CI: 8.14, 35.17, *p* = 0.002). RCS analysis confirmed a linear positive relationship. Subgroup analysis indicated a stronger association in non-drinkers (*p* for interaction < 0.05). Mediation analysis revealed that SII and SIRI partially mediated the relationship between DI-GM and serum Klotho, with mediation proportions of 7.15% and 5.68%, respectively.

**Conclusion:**

A higher DI-GM Score is significantly associated with increased serum Klotho levels among middle-aged and older adults, with systemic inflammation serving as a partial mediator. These findings suggest that dietary patterns promoting gut microbiota health may enhance Klotho levels, potentially contributing to anti-inflammatory and anti-aging processes in this population.

## 1 Background

Aging is a multifactorial process influenced by genetic, metabolic, and environmental factors. Among the molecular mediators implicated in this process, Klotho is a membrane-bound, soluble protein encoded by the Klotho gene, and has been identified as a key regulator of aging and longevity (1). Originally discovered in 1997 by Kuro-o et al. (2), Klotho plays a critical role in phosphate homeostasis, reduction of oxidative stress, and regulation of systemic inflammation. Reduced serum Klotho levels have been associated with age-related diseases, including chronic kidney disease (CKD), cardiovascular disease (CVD), and metabolic syndrome (3–6). Given its broad physiological significance, understanding the factors that influence Klotho expression and circulation is essential for developing potential therapeutic strategies to promote healthy aging.

In recent years, research has revealed the critical role of the gut microbiota in the biological aging process (7–9). With advancing age, the composition and function of the gut microbiota undergo significant changes, characterized by a decrease in beneficial bacteria and an increase in harmful species, leading to microbial dysbiosis. This imbalance is closely associated with hallmark features of aging, including chronic low-grade inflammation and impaired immune function (10, 11). Given the dynamic interplay between diet and the gut microbiome, the profound influence of diet on the composition and function of the gut microbiota has been widely recognized. To quantify this impact, researchers have proposed the Dietary Index for Gut Microbiota (DI-GM), which assesses the overall influence of dietary patterns on the gut microbiome. The DI-GM integrates both beneficial and harmful dietary components to comprehensively evaluate dietary quality and its potential role in modulating gut microbial diversity (12).

Chronic low-grade inflammation, commonly referred to as “inflammaging,” is considered a key factor contributing to the suppression of Klotho expression. Proinflammatory cytokines, such as tumor necrosis factor-α (TNF-α) and interleukin-6 (IL-6), have been shown to downregulate Klotho expression, thereby exacerbating age-related pathologies (13). In addition to cytokine-mediated effects, systemic oxidative stress, and immune dysregulation also contribute to Klotho suppression, highlighting the multifaceted mechanisms linking inflammation to the aging process (14).

Despite growing interest in the microbiota-aging axis, no study has yet explicitly examined the relationship between the Dietary Index for Gut Microbiota (DI-GM) and serum Klotho levels. This study aims to investigate the association between DI-GM and serum Klotho levels using data from the 2007–2016 National Health and Nutrition Examination Survey (NHANES). We hypothesize that a higher DI-GM, indicating a diet that promotes greater gut microbiota diversity and stability, is positively associated with serum Klotho concentrations. Furthermore, we propose that systemic inflammation serves as a key mediator in this relationship. By elucidating these associations, our study seeks to offer novel insights into diet-modifiable pathways related to aging and to propose potential strategies for promoting longevity through nutritional interventions.

## 2 Materials and methods

### 2.1 Data availability

In the United States, the Centers for Disease Control and Prevention (CDC) conducts a cross-sectional survey called the National Health and Nutrition Examination Survey (NHANES), which assesses the health and nutritional status of the population through in-person interviews and physical examinations. Participants provide written informed consent, complete a health interview at home, and then undergo physical examinations at mobile examination centers, where urine and blood samples are collected.

### 2.2 Study population

**Figure 1:**
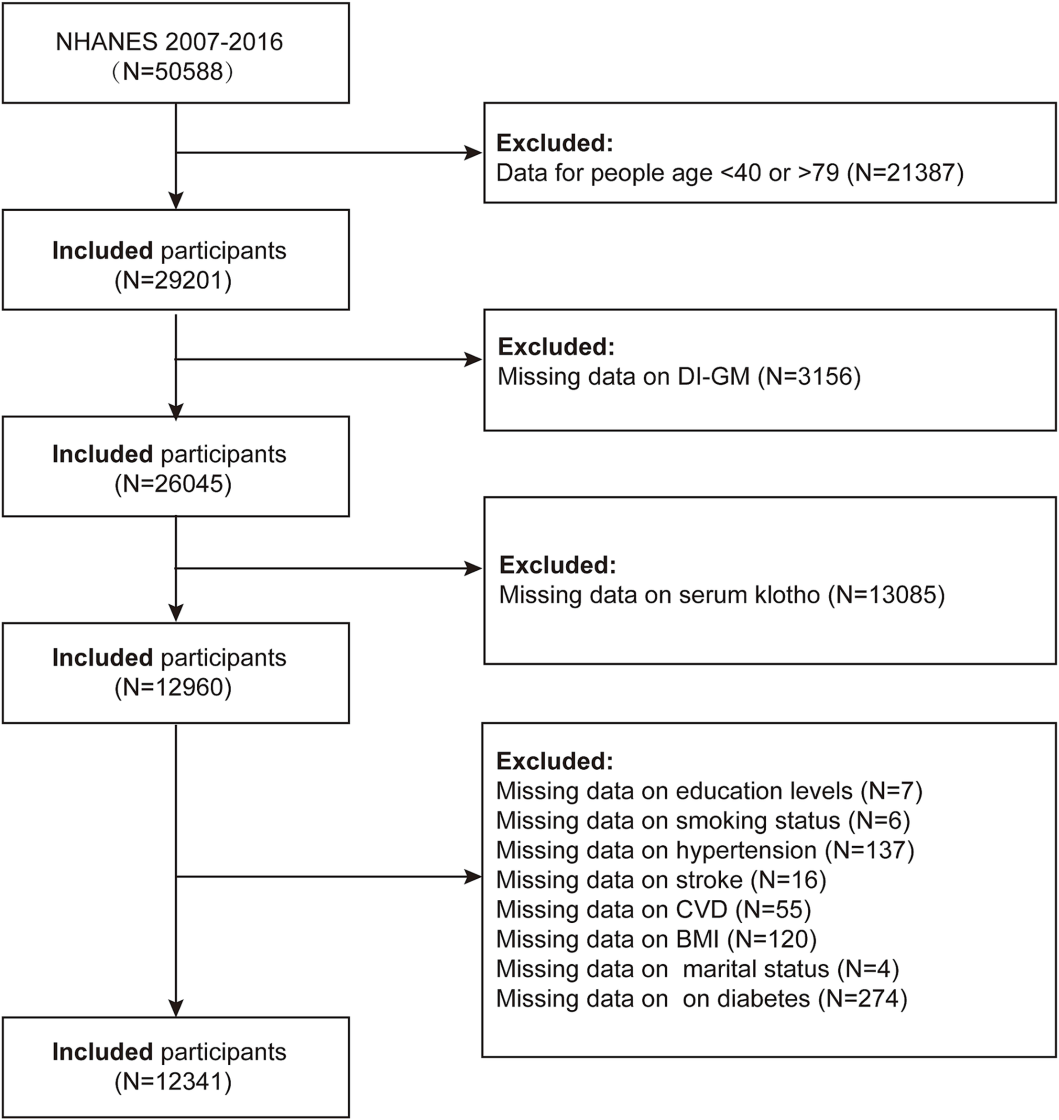
Flow chart of the sample selection from NHANES 2007–2016.

Our study utilized data from 2007 to 2016 to assess the association between the Dietary Index for Gut Microbiota (DI-GM) and serum Klotho levels, and to explore the potential mediating role of inflammatory responses. Initially, a total of 50,588 participants were included in the study. The exclusion criteria were as follows: (1) participants younger than 40 years or older than 79 years (*n* = 21,387); (2) participants with missing DI-GM data (*n* = 3,156); (3) participants with missing serum Klotho data (*n* = 13,085); (4) participants with missing education data (*n* = 7); (5) participants with missing smoking status (*n* = 6); (6) participants with missing hypertension status (*n* = 137); (7) participants with missing stroke status (*n* = 16); (8) participants with missing cardiovascular disease data (*n* = 55); (9) participants with missing body mass index (BMI) data (*n* = 120); (10) participants with missing marital status (*n* = 4); and (11) participants with missing diabetes status (*n* = 274). A total of 12,341 participants were included in the final analysis (Figure 1).

### 2.3 Definition of DI-GM

The Dietary Index for Gut Microbiota (DI-GM) Score is a dietary index designed to evaluate the impact of diet on gut microbiota health using 14 food and nutrient components, including 10 beneficial and four unfavorable dietary factors. Beneficial components, associated with improved gut microbiota composition, include fermented dairy, chickpeas, soybeans, whole grains, fiber, cranberries, avocados, broccoli, coffee, and green tea, while unfavorable components, which may negatively affect the gut microbiota, include red meat, processed meat, refined grains, and a high-fat diet (≥40% of total energy from fat) (12). The DI-GM score is calculated using 24-h dietary recall data from NHANES. For beneficial components, participants consuming ≥ the sex-specific median intake receive a score of 1, while those below the median receive a score of 0. Conversely, for unfavorable components, an intake ≥ the sex-specific median (or ≥40% for a high-fat diet) is assigned a score of 0, while a lower intake is assigned a score of 1. The total DI-GM score, ranges from 0 to 13, with higher scores indicating a diet more favorable to gut microbiota health. To facilitate analysis, the score is categorized into four groups: 0–3, 4, 5, and ≥6. Dietary intake data were collected through two 24-h dietary recall interviews in NHANES, with the first conducted in person at the Mobile Examination Center (MEC) and the second via telephone interview, ensuring a comprehensive assessment of participants’ dietary patterns (15). The specific scoring criteria for DI-GM can be found in Supplementary Table S1.

### 2.4 Definition of serum Klotho

Serum Klotho concentration was measured using a commercial ELISA kit (IBL International, Japan) following NHANES laboratory protocols. Frozen serum samples from individuals aged 40–79 years (NHANES 2007–2016) were stored at −80 °C before analysis and shipped to the Northwestern Lipid Metabolism and Diabetes Research Laboratory for processing. The assay sensitivity was 4.33 pg/mL, with a reference range of 285.8–1638.6 pg/mL (mean: 698.0 pg/mL). Each sample was analyzed in duplicate, with flagged discrepancies (>10% difference) requiring reanalysis. Quality control samples outside two standard deviations (2 SD) invalidated the test plate. Results were recorded in the Oracle Management System, ensuring compliance with NHANES quality assurance protocols (16, 17). See further details at: wwwn.cdc.gov/Nchs/Data/Nhanes/Public/2011/DataFiles/SSKL_G.htm.

### 2.5 Assessment of inflammatory markers

The Systemic Immune-Inflammation Index (SII) and Systemic Inflammation Response Index (SIRI) are CBC-derived inflammatory biomarkers calculated from peripheral blood cell counts. SII is defined as platelet count × neutrophil count/lymphocyte count, while SIRI is calculated as neutrophil count × monocyte count/lymphocyte count. These indices reflect systemic inflammatory and immune responses and were derived from complete blood count (CBC) measurements obtained using an automated hematology analyzer (Coulter® DxH 800), with values reported in ×10^3^ cells/μL (18, 19).

### 2.6 Covariates

In this study, covariates included sex, age, and race (Mexican American, other Hispanic, non-Hispanic White, non-Hispanic Black, and other races). Educational attainment was categorized as less than high school, high school, and more than high school. Marital status was classified as never married, married/living with a partner, and widowed/divorced/separated. The family income-to-poverty ratio (PIR) was divided into three categories: <1.3 (low income), 1.3–3.5 (middle income), and >3.5 (high income). Body mass index (BMI) was grouped as <25 kg/m^2^, 25–30 kg/m^2^, and ≥30 kg/m^2^. Smoking status was categorized into three groups: current smokers (smoked ≥100 cigarettes in the past year and currently smoking), former smokers (smoked ≥100 cigarettes in the past year but no longer smoking), and never smokers (smoked <100 cigarettes in the past year or never smoked). Alcohol drinkers were defined as individuals who drank more than 12 times in any given year of their life. Diabetes was defined as a self-reported physician diagnosis or a fasting blood glucose level ≥126 mg/dL. Hypertension was defined as a prior diagnosis, current use of antihypertensive medication, or an average of three blood pressure measurements ≥140/90 mmHg. Stroke was recorded as a binary variable (yes or no). Congestive heart failure, coronary heart disease, angina, or a history of myocardial infarction were considered indicators of cardiovascular disease. The reliability of these covariates has been supported by previous research (20).

### 2.7 Statistical analyses

Statistical analyses followed the analytical guidelines provided by NHANES, incorporating appropriate complex, multistage sampling weights. For continuous variables, survey-weighted means with 95% confidence intervals (CIs) were reported; for categorical variables, survey-weighted proportions with 95% CIs were presented. Weighted linear regression or weighted chi-square tests were used to assess differences across DI-GM quartiles.

Three weighted linear regression models were employed to examine the association between DI-GM and serum Klotho levels. Model 1 was unadjusted. Model 2 was adjusted for age, sex, and race/ethnicity. Model 3 was further adjusted for education, marital status, BMI, PIR, smoking status, alcohol consumption, diabetes, hypertension, cardiovascular disease, and stroke, in addition to the covariates included in Model 2. After covariate adjustment, restricted cubic spline (RCS) regression was used to evaluate potential nonlinear associations between DI-GM and serum Klotho levels. Finally, subgroup analyses and interaction tests were conducted based on potential confounders listed in the baseline characteristics to explore variations in associations across subpopulations.

Mediation analyses were conducted to assess the potential mediating effects of SII and SIRI on the relationship between DI-GM Score and serum Klotho levels. The Sobel test and bootstrap method with 1,000 simulations were used to estimate the 95% confidence intervals of the mediation effect. The mediation effect was quantified as the proportion mediated, calculated using the formula: (indirect effect/total effect) × 100%. Statistical significance of the mediation pathway was determined through bootstrap resampling, ensuring robust estimation of the indirect effects of SII and SIRI in linking DI-GM to serum Klotho levels. Statistical analyses were performed using R software (version 4.3.3), with statistical significance set at *p* < 0.05.

## 3 Results

### 3.1 Baseline characteristics of the study population

Table 1 summarizes the baseline characteristics of participants stratified by DI-GM score. A total of 12,341 participants were included in this study, with a mean age of 56.16 years; 47.68% were male, and the average serum Klotho level was 838.13 pg/mL. Significant age differences were observed across the DI-GM groups (*p* = 0.0096), with the ≥6 score group having the highest mean age. A higher proportion of males was found in the 0–3 score group, whereas more females were observed in the ≥6 score group. Race and educational level were significantly associated with DI-GM scores, with non-Hispanic White person and individuals with higher education more commonly represented in the ≥6 score group (*p* < 0.001). Married or cohabiting individuals were more prevalent in the ≥6 score group. Higher-income was associated with higher DI-GM scores. The 0–3 score group had a higher prevalence of obesity and smoking, whereas the ≥6 score group had a greater proportion of participants with a normal BMI (*p* < 0.001). Hypertension, diabetes, and cardiovascular disease were more prevalent among participants with lower DI-GM scores (all *p* < 0.05) (Table 1).

**Table 1:** Baseline table for population based on DI-GM score.

Characteristics	Total (*n* = 12,341)	DI-GM				*p*-value
		0–3 score (*n* = 2,138)	4 score (*n* = 2,473)	5 score (*n* = 2,811)	≥6 score (*n* = 4,919)	
Age (years)	56.16 (55.86,56.47)	55.94 (55.33,56.55)	55.55 (54.99,56.11)	55.99 (55.43,56.56)	56.58 (56.15,57.00)	0.0096
Gender (%)						
Male	47.68 (46.82,48.54)	54.24 (50.75,57.69)	51.81 (49.58,54.04)	45.38 (42.89,47.88)	44.91 (43.34,46.50)	<0.0001
Female	52.32 (51.46,53.18)	45.76 (42.31,49.25)	48.19 (45.96,50.42)	54.62 (52.12,57.11)	55.09 (53.50,56.66)	
Race (%)						
Mexican American	6.52 (5.17,8.20)	7.17 (5.40,9.46)	8.06 (6.26,10.33)	7.37 (5.70,9.47)	5.25 (4.17,6.59)	<0.0001
Other Hispanic	4.59 (3.66,5.75)	5.40 (3.95,7.34)	6.08 (4.66,7.88)	4.92 (3.81,6.32)	3.55 (2.88,4.38)	
Non-Hispanic White	74.03 (70.94,76.89)	68.15 (63.95,72.07)	69.03 (64.77,72.99)	72.61 (68.99,75.96)	78.79 (76.08,81.27)	
Non-Hispanic Black	9.01 (7.65,10.58)	14.61 (12.24,17.35)	11.42 (9.32,13.93)	8.75 (7.34,10.40)	6.23 (5.24,7.40)	
Other race	5.85 (5.10,6.70)	4.67 (3.64,5.97)	5.41 (4.27,6.83)	6.35 (5.22,7.71)	6.17 (5.25,7.24)	
Education level (%)						
Less than high school	16.02 (14.44,17.75)	20.54 (18.15,23.16)	20.74 (18.22,23.49)	15.90 (14.09,17.90)	12.61 (10.85,14.62)	<0.0001
High school	22.15 (20.82,23.53)	29.75 (27.22,32.41)	24.73 (21.87,27.82)	22.96 (20.50,25.62)	18.08 (16.48,19.80)	
More than high school	61.83 (59.50,64.11)	49.71 (46.55,52.87)	54.54 (50.79,58.23)	61.13 (57.87,64.30)	69.31 (66.55,71.94)	
Marital status (%)						
Never married	7.24 (6.46,8.11)	8.39 (6.76,10.38)	7.09 (5.95,8.43)	6.89 (5.55,8.52)	7.09 (6.05,8.29)	0.0012
Married/living with partner	70.63 (69.10,72.11)	67.17 (64.00,70.20)	67.98 (65.54,70.32)	70.84 (68.43,73.13)	72.79 (70.68,74.80)	
Widowed/divorced/separated	22.13 (20.93,23.37)	24.43 (21.80,27.27)	24.93 (22.83,27.17)	22.27 (20.37,24.30)	20.13 (18.59,21.75)	
PIR (%)						
<1.3	17.86 (16.18,19.67)	23.76 (21.36,26.34)	23.21 (20.94,25.66)	19.73 (17.42,22.25)	12.72 (11.17,14.44)	<0.0001
1.3–3.5	32.06 (30.33,33.84)	33.80 (30.66,37.08)	35.27 (32.56,38.08)	31.97 (29.05,35.05)	30.21 (27.98,32.54)	
≥3.5	50.08 (47.25,52.90)	42.44 (38.40,46.59)	41.52 (37.98,45.15)	48.30 (44.56,52.06)	57.07 (53.85,60.24)	
BMI (%)						
<25	24.52 (23.30,25.78)	19.83 (17.55,22.33)	22.32 (19.95,24.88)	23.69 (21.47,26.06)	27.44 (25.62,29.33)	<0.0001
25–30	35.13 (33.97,36.30)	33.07 (30.58,35.66)	35.48 (32.72,38.34)	34.80 (32.47,37.21)	35.85 (33.98,37.77)	
≥30	40.35 (38.89,41.83)	47.10 (44.33,49.89)	42.20 (39.49,44.97)	41.51 (39.35,43.69)	36.71 (34.59,38.88)	
Smoking status (%)						
Never	51.76 (50.38,53.13)	49.64 (46.47,52.82)	50.84 (47.89,53.78)	50.55 (47.87,53.23)	53.47 (51.68,55.25)	<0.0001
Now	18.20 (17.02,19.45)	22.82 (20.16,25.72)	20.85 (18.47,23.46)	19.18 (17.25,21.27)	15.05 (13.68,16.52)	
Former	30.04 (28.74,31.37)	27.53 (25.14,30.06)	28.31 (25.72,31.04)	30.27 (27.77,32.90)	31.48 (29.55,33.48)	
Alcohol intake (%)						
No	1.00 (0.77,1.31)	0.86 (0.43,1.72)	0.87 (0.45,1.67)	0.85 (0.45,1.62)	1.19 (0.83,1.70)	0.6712
Yes	99.00 (98.69,99.23)	99.14 (98.28,99.57)	99.13 (98.33,99.55)	99.15 (98.38,99.55)	98.81 (98.30,99.17)	
Hypertension (%)						
No	51.72 (50.31,53.12)	46.48 (43.70,49.27)	49.18 (46.38,51.98)	51.48 (48.76,54.20)	54.66 (52.52,56.78)	<0.0001
Yes	48.28 (46.88,49.69)	53.52 (50.73,56.30)	50.82 (48.02,53.62)	48.52 (45.80,51.24)	45.34 (43.22,47.48)	
Diabetes (%)						
No	82.17 (81.00,83.29)	76.84 (73.71,79.70)	78.27 (76.14,80.25)	82.80 (80.52,84.87)	85.27 (84.06,86.41)	<0.0001
Yes	17.83 (16.71,19.00)	23.16 (20.30,26.29)	21.73 (19.75,23.86)	17.20 (15.13,19.48)	14.73 (13.59,15.94)	
Stroke (%)						
No	96.78 (96.42,97.11)	96.41 (95.24,97.30)	95.99 (95.00,96.79)	96.81 (95.93,97.50)	97.22 (96.61,97.72)	0.1148
Yes	3.22 (2.89,3.58)	3.59 (2.70,4.76)	4.01 (3.21,5.00)	3.19 (2.50,4.07)	2.78 (2.28,3.39)	
CVD (%)						
No	91.28 (90.65,91.88)	90.86 (89.51,92.05)	89.74 (88.45,90.91)	91.07 (89.58,92.37)	92.16 (91.21,93.02)	0.0163
Yes	8.72 (8.12,9.35)	9.14 (7.95,10.49)	10.26 (9.09,11.55)	8.93 (7.63,10.42)	7.84 (6.98,8.79)	
SII	546.95 (537.50,556.40)	569.61 (543.83,595.39)	552.33 (538.48,566.18)	553.12 (537.36,568.88)	533.90 (522.89,544.91)	0.0119
SIRI	1.28 (1.26,1.31)	1.36 (1.31,1.41)	1.32 (1.28,1.37)	1.29 (1.25,1.34)	1.23 (1.20,1.26)	0.0001
Klotho (pg/mL)	838.13 (828.99,847.27)	815.55 (800.56,830.54)	837.41 (824.07,850.76)	834.27 (819.64,848.89)	848.10 (835.08,861.12)	0.0071

PIR, poverty income ratio; BMI, body mass index; CVD, cardiovascular disease; DI-GM, dietary index for gut microbiota; SII, systemic immune-inflammation index; SIRI, system inflammation response index.

### 3.2 Association between DI-GM and serum Klotho

**Figure 2:**
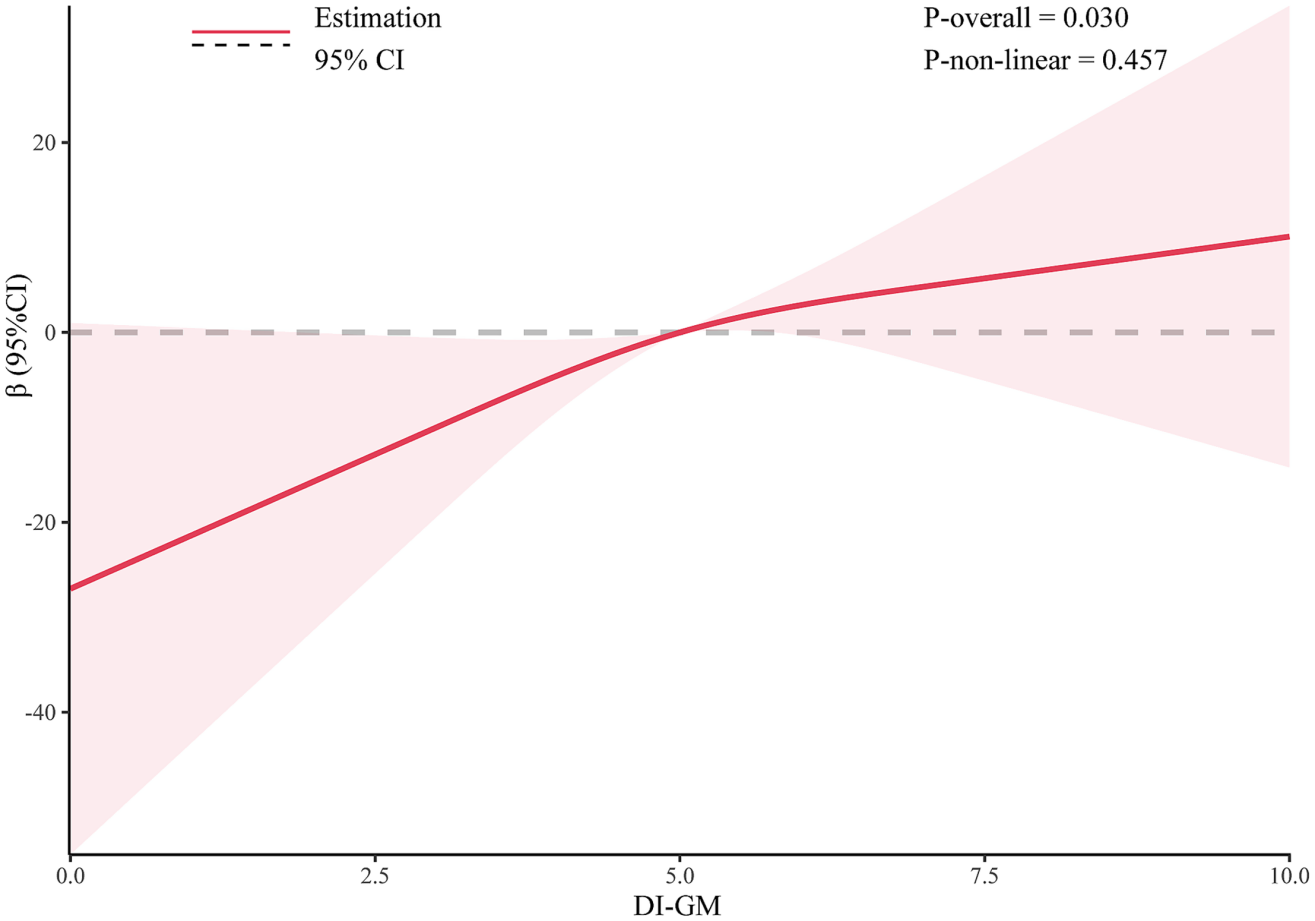
The dose-effect relationship between DI-GM and serum Klotho. Gender, age, race, education level, marital status, PIR, BMI, smoking status, alcohol consumption, diabetes, hypertension, CVD, and stroke were adjusted.

Results from multivariable linear regression analysis demonstrated a positive association between DI-GM and serum Klotho levels. After adjusting for all covariates, DI-GM remained significantly positively associated with serum Klotho (*β* = 4.05; 95% CI: 1.31, 6.80). Each 1 score increase in DI-GM was associated with a 4.05 pg/mL increase in serum Klotho concentration. Additionally, DI-GM was transformed from a continuous to a categorical variable. Compared with participants in the lowest quartile, those with a DI-GM score ≥6 had significantly higher serum Klotho levels (β = 21.65; 95% CI: 8.14, 35.17), with a significant trend across categories (Table 2). Furthermore, restricted cubic spline (RCS) analysis indicated a linear positive association between DI-GM and serum Klotho levels (Figure 2).

**Table 2:** Multivariate linear regression analysis of DI-GM and serum Klotho.

Characteristic	Model 1		Model 2		Model 3	
	*β* (95% CI)	*p-*value	*β* (95% CI)	*p-*value	*β* (95% CI)	*p-*value
DI-GM (continuous)	3.98 (1.31, 6.66)	0.004	5.29 (2.60, 7.98)	<0.001	4.05 (1.31, 6.80)	0.004
DI-GM (group)						
0–3	Reference		Reference		Reference	
4	13.03 (−2.22, 28.28)	0.094	13.60 (−1.52, 28.73)	0.078	12.04 (−3.04, 27.12)	0.118
5	15.62 (0.80, 30.43)	0.039	17.10 (2.37, 31.83)	0.023	14.86 (0.15, 29.57)	0.048
≥6	21.42 (8.05, 34.80)	0.003	26.71 (13.32, 40.09)	<0.001	21.65 (8.14, 35.17)	0.002
*p* for trend		<0.001		<0.001		<0.001

Model 1: No covariates were adjusted. Model 2: Adjusted for age, gender, and race. Model 3: Adjusted for age, gender, race, education level, marital status, PIR, BMI, smoking status, alcohol consumption, diabetes, hypertension, CVD, and stroke.

### 3.3 Subgroup analyses

**Figure 3:**
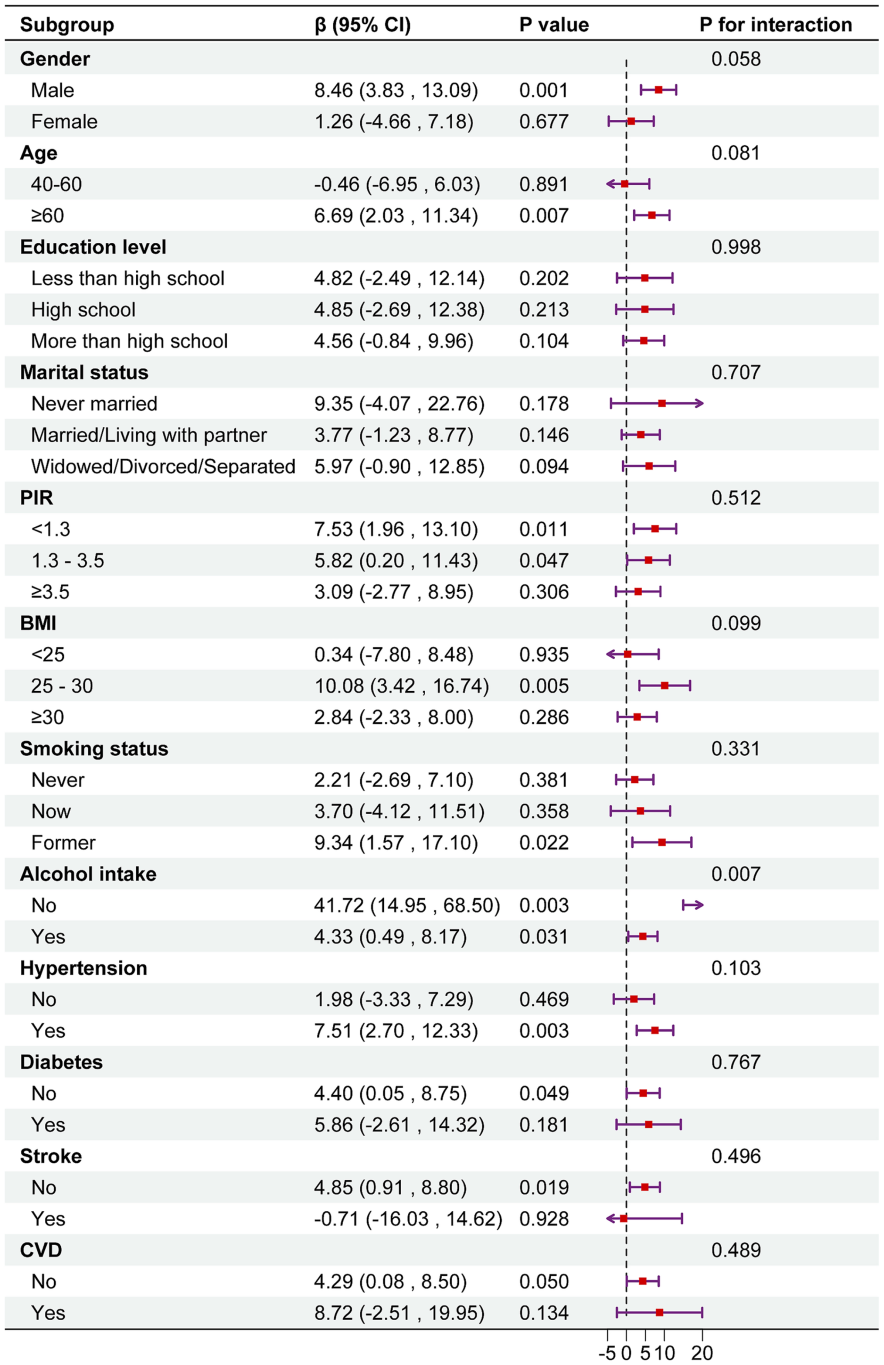
Subgroup analysis between DI-GM and serum Klotho. Note 1: The above model was adjusted for gender, age, race, education level, marital status, PIR, BMI, smoking status, alcohol consumption, diabetes, hypertension, CVD, and stroke. Note 2: In each case, the model was not adjusted for the stratification variable.

The results indicated that the positive association between DI-GM and serum Klotho levels was consistently observed across all subgroups. Interestingly, a significant interaction was observed in the subgroup analysis by alcohol consumption status (*p* for interaction <0.05). The association between DI-GM and serum Klotho was stronger among non-drinkers (*β* = 41.72; 95% CI: 14.95, 68.50) (Figure 3).

### 3.4 Mediating analysis

**Figure 4:**
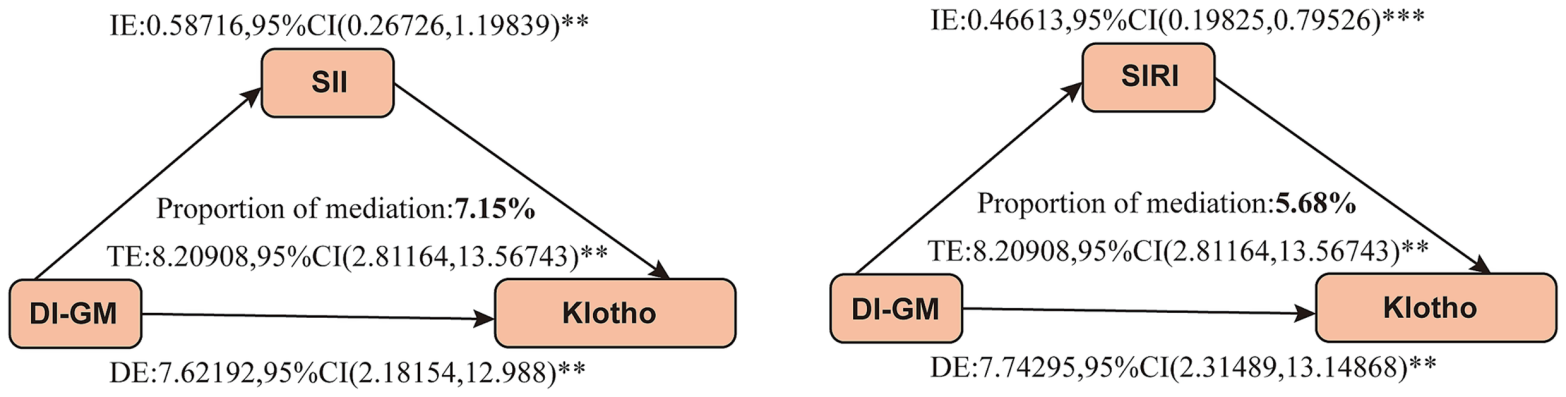
Mediation analysis between DI-GM and serum Klotho levels. Gender, age, race, education level, marital status, PIR, BMI, smoking status, alcohol consumption, diabetes, hypertension, CVD, and stroke were adjusted. DI-GM, dietary index for gut microbiota; SII, systemic immune-inflammation index; SIRI, system inflammation response index; TE, total effect; IE, indirect effect; DE, direct effect; **p < 0.05, ***p < 0.001. *p* < 0.05 suggests significant differences.

We further conducted a mediation analysis to investigate the mediating role of inflammatory markers in the association between DI-GM and serum Klotho levels. The results indicated that both SII and SIRI exerted significant mediating effects, with mediation proportions of 7.15% and 5.68%, respectively (Figure 4).

## 4 Discussion

This study provides novel evidence linking the Dietary Index for Gut Microbiota (DI-GM) to serum Klotho levels and highlights the mediating role of systemic inflammation. Our findings suggest that higher DI-GM scores—indicative of dietary patterns that promote gut microbiota health—are significantly associated with increased serum Klotho levels. In addition, both SII and SIRI were found to partially mediate this association, suggesting that dietary modulation of the gut microbiota may influence systemic inflammation and aging-related biomarkers. These results advance our understanding of how diet-driven microbial changes translate into molecular markers of aging, thereby contributing to the growing body of research on diet–microbiota interactions and their impact on aging and metabolic regulation.

Our findings are consistent with previous studies, underscoring the pivotal role of the gut microbiota in aging and metabolic health. In the present study, a higher DI-GM score was significantly associated with elevated serum Klotho levels. This observation is in line with prior evidence suggesting that diet-induced microbial changes may influence host aging pathways. For instance, studies have shown that high dietary fiber intake increases the abundance of *Bifidobacterium* and *Faecalibacterium prausnitzii*, and the enrichment of these beneficial microbes has been shown to alleviate gut inflammation in aged mice (21). In addition, a cross-sectional study by Wu et al. (22) reported that adherence to the Mediterranean diet was significantly associated with higher serum Klotho levels. Another study demonstrated that higher DI-GM scores were associated with a lower risk of accelerated aging, with BMI acting as a mediator of this relationship (23). A Mendelian randomization study provided genetic evidence supporting a causal relationship between specific gut microbial taxa and accelerated biological aging, highlighting the gut microbiota as a potentially modifiable target for aging-related interventions (24).

Emerging evidence suggests that dietary intake has a profound impact on the composition and function of the gut microbiota, thereby influencing inflammation and metabolic health. High-fiber diets, polyphenol-rich foods, and fermented products enhance microbial diversity and promote the production of short-chain fatty acids (SCFAs), bioactive metabolites that have been associated with reduced inflammation and improved insulin sensitivity (25). In contrast, diets rich in processed meats, refined grains, and high-fat foods can induce microbial dysbiosis, leading to increased gut permeability and systemic inflammation (26). Our findings support these observations, showing that individuals with higher DI-GM scores had lower levels of SII and SIRI, which partially explains the positive association between DI-GM and serum Klotho levels.

Chronic inflammation is considered a hallmark of aging and is implicated in the pathogenesis of various chronic diseases, including cardiovascular disease, chronic kidney disease (CKD), and neurodegenerative disorders. Klotho, in contrast, exhibits anti-inflammatory and cytoprotective properties (27, 28). Previous studies have shown that Klotho deficiency leads to the upregulation of inflammatory cytokines such as IL-6 and TNF-α, whereas exogenous Klotho supplementation reduces systemic inflammation (13). Moreover, studies have reported that in CKD patients, lower Klotho levels are often accompanied by elevated CRP and IL-6 levels, further supporting the anti-inflammatory role of Klotho (29). Other research has indicated that the Dietary Inflammatory Index (DII) is negatively associated with both telomere length and Klotho levels (30). In this study, we used SII and SIRI as markers of systemic inflammation, whereas most prior studies have primarily relied on CRP or IL-6. SII and SIRI integrate multiple hematological parameters, including neutrophils, lymphocytes, and platelets, which may provide a more comprehensive reflection of systemic inflammation and offer greater sensitivity than single biomarkers. Interestingly, subgroup analysis revealed a stronger association between DI-GM and Klotho among non-drinkers. This finding is consistent with previous studies showing that alcohol consumption alters gut microbiota composition, increases intestinal permeability, and promotes systemic inflammation (31, 32). Alcohol-induced dysbiosis may counteract the beneficial effects of high DI-GM diets on inflammation and Klotho expression, warranting further investigation.

The potential mechanisms underlying our findings involve complex interactions among diet, the gut microbiota, systemic inflammation, and the regulation of Klotho expression. The gut microbiota modulates systemic inflammation through multiple pathways, including the production of SCFAs, maintenance of intestinal barrier integrity, and regulation of immune responses (33). SCFAs such as butyrate and propionate exert anti-inflammatory effects by inhibiting NF-κB signaling and promoting the differentiation of regulatory T cells, thereby reducing systemic inflammation and oxidative stress (34, 35). These effects may facilitate Klotho expression, as inflammation-induced oxidative stress has been shown to suppress Klotho gene transcription (36).

Our findings provide a foundation for future studies to further elucidate the causal pathways linking diet, gut microbiota, systemic inflammation, and Klotho expression. Prospective cohort studies are warranted to establish temporality and assess whether changes in DI-GM precede alterations in Klotho levels. Randomized dietary intervention trials incorporating DI-GM-based modifications, combined with microbiome and metabolomic profiling, could help identify specific dietary components and microbial taxa responsible for Klotho regulation. In addition, mechanistic studies at the cellular and molecular levels are needed to confirm the role of inflammation and other pathways, such as oxidative stress modulation, in mediating these effects. From a public health perspective, promoting dietary patterns that enhance gut microbial diversity and metabolic output could represent a feasible and non-pharmacological strategy for supporting healthy aging and mitigating inflammation-related chronic diseases. This may be particularly relevant in aging populations and in individuals at risk for metabolic or inflammatory disorders, where dietary interventions could be implemented as part of lifestyle-based preventive programs.

## 5 Strengths and limitations

This study has several strengths. First, to our knowledge, this is the first study to comprehensively examine the association between DI-GM and serum Klotho levels using a large, nationally representative NHANES dataset. The large sample size and population-based design enhance the generalizability of our findings. Second, we employed robust statistical methods to rigorously evaluate the potential relationships among DI-GM, systemic inflammation, and serum Klotho. Third, compared with single inflammatory markers such as CRP or IL-6, the use of SII and SIRI provides a more comprehensive assessment of systemic inflammation. These composite indices, which integrate multiple immune cell counts, may therefore serve as more reliable indicators of overall immune status.

Despite these strengths, several limitations should be acknowledged. First, due to the cross-sectional nature of NHANES, we were unable to establish causal relationships among DI-GM, systemic inflammation, and serum Klotho levels. Although our mediation analysis suggested a potential pathway, longitudinal studies and randomized controlled trials are needed to confirm the directionality of these associations. Second, although DI-GM is a useful dietary index reflecting microbiota-related dietary patterns, it does not directly measure the composition or function of the gut microbiota. Future investigations integrating gut microbiome sequencing and metabolomic profiling are essential to identify the specific microbial taxa and metabolites that mediate the DI-GM–Klotho relationship. Third, although we adjusted for a wide range of potential confounders, residual confounding cannot be entirely ruled out. Thus, prospective cohort studies with repeated measures are warranted to better elucidate temporal relationships and strengthen causal inference. Moreover, personalized nutrition strategies tailored to individual gut microbiota profiles should also be explored to optimize dietary interventions that promote healthy aging and longevity.

## 6 Conclusion

Our findings indicate that a higher DI-GM score is significantly associated with elevated serum Klotho levels, with systemic inflammation acting as a partial mediator. This suggests that dietary patterns beneficial to gut microbiota may contribute to increased Klotho expression and reduced systemic inflammation, potentially supporting healthy aging and metabolic regulation. These results highlight the importance of diet-driven gut microbiota modulation in influencing longevity-related biomarkers. Further research is warranted to elucidate the underlying mechanisms and explore dietary interventions that optimize gut microbiota composition for improved systemic health.

## Data Availability

The original contributions presented in the study are included in the article/Supplementary material, further inquiries can be directed to the corresponding authors.
